# Survey of *in vitro* fertilization add-ons in Japan (Izanami project)

**DOI:** 10.3389/fendo.2024.1404601

**Published:** 2024-10-08

**Authors:** N. Shionoya, M. Yamada, S. Harada, H. Shirasawa, S. Chik Jwa, K. Kuroda, M. Harada, Y. Osuga

**Affiliations:** ^1^ Department of Obstetrics and Gynecology, Keio University School of Medicine, Tokyo, Japan; ^2^ Department of Obstetrics and Gynecology, Akita University Graduate School of Medicine, Akita, Japan; ^3^ Department of Obstetrics and Gynecology, Saitama Medical University, Saitama, Japan; ^4^ Center for Reproductive Medicine and Endoscopy, Sugiyama Clinic Marunouchi, Tokyo, Japan; ^5^ Department of Obstetrics and Gynecology, Graduate School of Medicine, The University of Tokyo, Tokyo, Japan

**Keywords:** adoption, cost, *in vitro* fertilization, add-ons, Japan

## Abstract

**Objective:**

To identify any correlations between evidence levels, adoption rates, and additional costs of *in vitro* fertilization (IVF) add-ons.

**Design:**

Online survey.

**Subjects:**

The survey was conducted in 621 assisted reproductive technology-registered facilities that are members of the Japanese Society of Obstetrics and Gynecology from December 22, 2021, to February 13, 2022.

**Exposure:**

The survey included details regarding the specific add-on modalities employed and their associated costs; inquiries pertained to the fertility healthcare infrastructure in Japan before the implementation of the National Health Insurance scheme.

**Main outcome measures:**

The correlation between the adoption rate and cost of IVF add-ons and their evidence levels were analyzed. The evidence level of the add-on treatments was classified into Green, Amber, and Red categories based on the United Kingdom’s Human Fertilisation and Embryology Authority and Cochrane systematic reviews.

**Results:**

A total of 438 eligible responses were analyzed, with clinics constituting 70.9% of the respondents’ facilities. A total of 18 add-ons were assessed, and 96.5% (423/438) of facilities used at least one add-on. A positive correlation of the adoption rate and an inverse correlation of the cost with the evidence level of the IVF add-on treatment were observed (not significant). Outpatient clinics, defined as medical facilities with no beds, had a significantly higher adoption rate (Amber, 65.7%; Red, 52.0%) of add-ons than other facilities, regardless of the evidence rating, although the costs were similar across all site attributes.

**Conclusion:**

Accumulating evidence on the efficacy and safety of add-ons will lead to the development of medical care with a high-cost benefit, as an increase in the adoption rate and a decrease in cost are expected when limiting to medical care with a high level of evidence.

## 1 Introduction

Routine cycles of proven fertility treatment are effective without the addition of unproven treatment add-ons. Assisted reproductive technology (ART) is considered the most effective method for treating infertility, although it has been reported that only approximately 60%–70% of couples who undergo ART are able to achieve live births (1), indicating the existence of limitations in the therapeutic approach. A study conducted by the United Kingdom’s Human Fertilisation and Embryology Authority (HFEA) revealed that 74% of patients undergoing medical treatment for infertility utilized “*in vitro* fertilization (IVF) add-on” treatments, with the number of patients using them increasing yearly (2). An agreement between the HFEA and other professional and patient groups (Consensus Statement, October 19, 2023) states that treatments without strong evidence of safety and/or efficacy should only be offered in research settings. According to a survey conducted in Australia, the most frequently used “add-on” treatments are preimplantation genetic testing for aneuploidy (PGT-A) (27%), time-lapse technology (TLT) (23%), hyaluronic acid-containing culture media (22%), and assisted hatching (AHA) (8%) (3).

Despite the lack of scientific evidence, “IVF add-on” treatments are widely used and have become an international issue, owing to the high financial burden placed on patients (4). In Japan, some add-ons, such as artificial oocyte activation (AOA), AHA, and Hyaluronate, have been made eligible for national health insurance coverage from 2022 onward. When introducing “add-on” treatments, medical professionals are required to explain their effectiveness and safety and obtain informed consent. However, it is suspected that many patients undergo add-on treatments without sufficient explanation, which could increase the likelihood of patient regret. In a survey conducted by Lensen et al., the percentage of regret increased when the patient’s role in the decision to use the selected add-on was <50% (5). Furthermore, when medical professionals fail to provide sufficient information regarding the evidence level and details of the treatment, patients may rely on misinformation from external sources and experience regret when the therapeutic approach fails to yield positive results.

To alleviate the burden on patients, it is essential to clarify the clinical status of add-on treatments. The Japanese government began providing national insurance coverage for ART in 2022. However, little information is available regarding the adoption rates and additional costs of IVF add-ons at IVF centers. Therefore, to gain insight into the medical system and reality of add-on treatments before insurance coverage, we conducted a survey to establish evidence for the introduction of new therapeutic approaches and to determine the essential medical systems required for this purpose.

## 2 Materials and methods

### 2.1 Methods and timelines of the survey

We conducted an empirical survey, named the IZANAMI survey project (toward the Introduction of new technologies for handling Zygotes Survey on treatment ADD-ONS and Assisted Reproductive MedIcine in Japan), targeting 616 ART facilities, after modifying for closures/integrations (five facilities), cessation of handling of IVF (three facilities), and additional/newly established facilities (three facilities), based on 621 ART facilities registered with the Japan Society of Obstetrics and Gynaecology. We conducted the survey using a Google Form (December 22, 2021, to February 13, 2022) (6). Of the 478 responses obtained, 41 duplicate responses were removed; therefore, 437 responses were included (response rate, 70.9%). Based on the responses obtained from the target facilities, we analyzed the implementation status and cost of IVF add-on treatments in Japan.

### 2.2 Adoption rate of IVF add-on treatment

In Japan, medical institutions are classified based on the number of beds they have. According to the Japanese Medical Care Act, a clinic is defined as a medical facility with 19 or fewer beds, distinguishing it from a hospital, which must have 20 or more beds. A unique feature of the Japanese healthcare system is the prevalence of clinics without any inpatient beds, which focus entirely on outpatient care. These no-bed clinics play a significant role in providing accessible medical services, particularly in urban areas where space is limited and outpatient care is in high demand. Accordingly, each facility was defined and consistently referred to as “outpatient clinics (no bed),” “inpatient clinics (19 or fewer beds),” “hospital (more than 20 beds),” or “university hospital.”.

Outpatient clinics are considered dominant because they handle the largest number of oocyte retrieval cycles and account for approximately half of all the facilities in Japan. Accordingly, we examined variations in add-ons using outpatient clinics as controls. Add-on treatments with adoption rates that are more than twice as high between facility types compared with outpatient clinics were defined as “variations” and analyzed accordingly.

### 2.3 Evidence level rating of IVF add-ons

In addition to add-ons listed in the HFEA, we included those identified by a Japanese Ministry of Health, Labour and Welfare survey (7). We independently classified the add-on treatments into Green, Amber, and Red categories, based on the level of evidence of clinical effectiveness currently available in the HFEA (8) and Cochrane systematic reviews. No add-ons were classified as the Green category (where there is more than one high quality randomized controlled trial [RCT] which shows that the procedure is effective at improving live birth rate for most fertility patients). The add-ons included in the Amber category (where there is conflicting evidence from RCTs to show that an add-on is effective at improving live birth rate for most fertility patients) were AOA, hyaluronic acid-containing culture media, and TLT. The add-ons included in the Red category (no RCT studies have shown an effect on improving the chances of having a child for most infertile patients) were AHA, endometrial receptivity analysis (ERA), interferon-γ-producing helper-T cell (Th1)/IL-4-producing helper-T cell (Th2) ratio test, intracytoplasmic morphologically selected sperm injection (IMSI) (9), and PGT-A. The evidence level ratings of add-ons, including AHA (10), hyaluronic acid-containing culture media (11), endometrial injection of embryo culture supernatant (12), immunosuppressant agent treatment (13), PGT-A (14), and granulocyte colony-stimulating factor infusion (15) were verified based on Cochrane systematic reviews. Add-ons not addressed by either HFEA or Cochrane (testing and treatment of chronic endometritis, including ERA, endometrial microbiome metagenomic analysis [EMMA], two-step embryo transfer, platelet-rich plasma [PRP] intrauterine injection, and *in vitro* maturation [IVM]) were also classified as Red. The Cochrane review (16) updated the recommendations for endometrial scratch and included the Lensen 2019 trial, which found no significant difference between endometrial scratch (n = 690, live birth rate 26.1% [180/690]) and controls (n = 674, live birth rate 26.1% [176/674]) (odds ratio, 1.00 [95% confidence interval, 0.78-1.27]) (5). Accordingly, in the current study, we decided to classify endometrial scratch as Red instead of Amber.

### 2.4 Statistical analyses

We performed statistical analysis by conducting a t-test using the JMP software program (JMP^®^, Version 15; SAS Institute Inc., Cary, NC, USA) to examine the adoption rate and cost among facilities in relation to IVF add-ons. A total of 208 outpatient clinics, accounting for 47% of all facilities, were used as controls. Facilities that utilized an add-on but did not provide information on costs were excluded from the analysis. The following notations were adopted:

1. The adoption rate of each add-on medical treatment was indicated in the order of the number of facilities implementing it/total number of facilities × 100 (%).
2. The cost for each add-on medical treatment was indicated as the median cost for each add-on (interquartile range).

### 2.5 Ethics

This study was a survey of medical facilities and was not suited to the “Ethical Guidelines for Medical and Health Research Involving Human Subjects.” Therefore, the requirement for ethics approval was waived by the local ethics committee.

## 3 Results

### 3.1 Facility types

**Figure 1:**
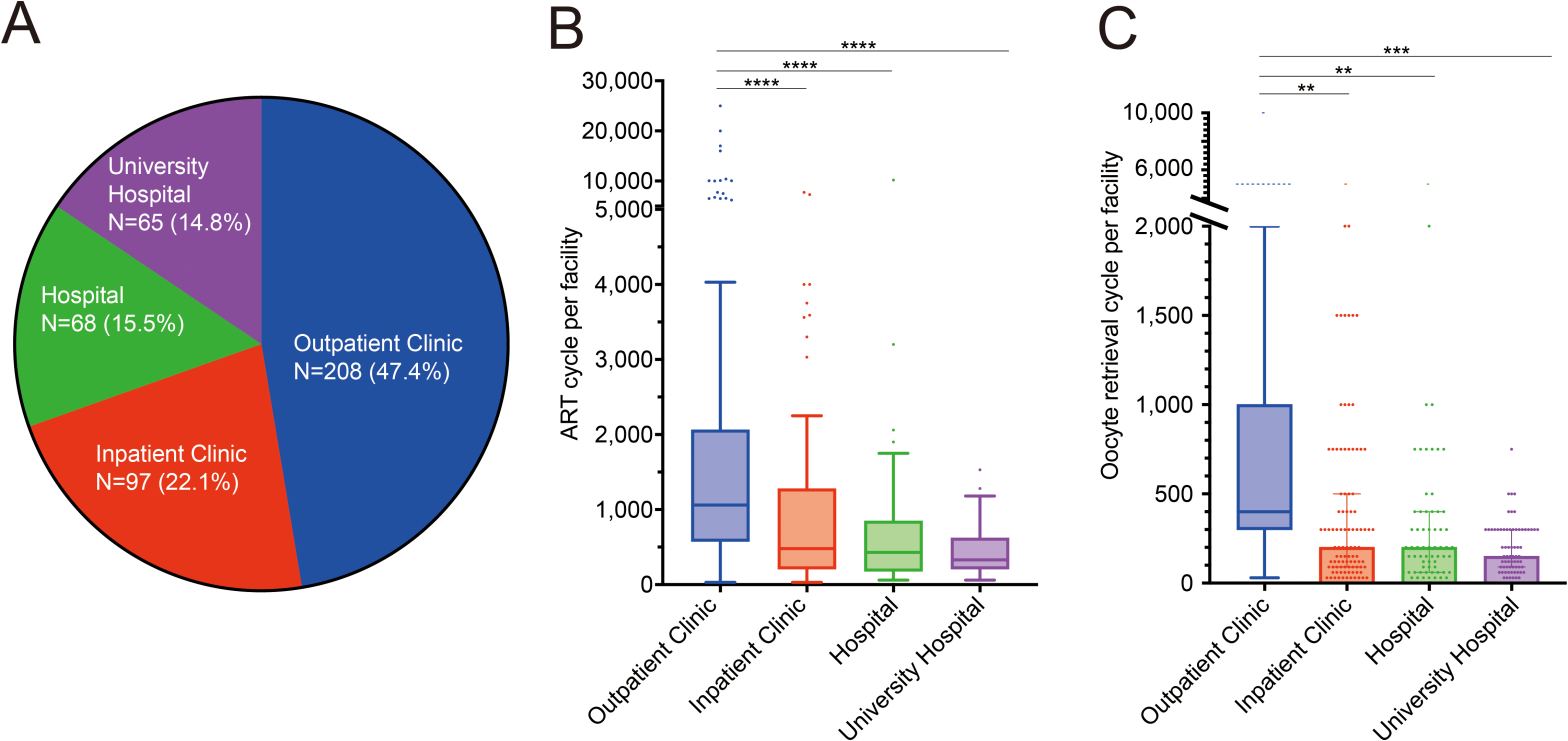
Facilities treating assisted reproductive technology in Japan. **(A)** Proportion of *in vitro* fertilization facilities in Japan. **(B, C)** Number of assisted reproductive technology cycles: total number of oocyte retrievals and embryo transfers per facility **(B)** and oocyte retrieval cycles **(C)**. ****p<0.0001, ***p<0.001, **p<0.01.

Facilities were categorized as outpatient clinics, inpatient clinics, hospitals, and university hospitals. Outpatient clinics accounted for the largest proportion, followed by hospital-based clinics and university hospitals. Clinics accounted for 69.5% of the total number of facilities (**Figure 1A**). The numbers of ART cycles (**Figure 1B**) and oocyte retrieval cycles (**Figure 1C**) were significantly higher in outpatient clinics than in other types of facilities.

### 3.2 Adoption rate of IVF add-on treatment

Add-on treatments with high adoption rates of >50% across all facility types were AHA, chronic endometritis testing and treatment, and AOA, whereas those with low adoption rates of <30% across all facility types were PGT-A, IVM, granulocyte colony-stimulating factor infusion, and IMSI (**Table 1**).

**Table 1:** Adoption rate of *in vitro* fertilization add-on treatments according to facility.

	Rating in the current study	Total	Outpatient Clinic	Inpatient Clinic	Hospital	University Hospital	
		(n=438)	(n=208)	(n=97)	(n=68)	(n=65)	*p* value
**Artificial Oocyte Activation (AOA)**	Amber	295 (67.3%)	167 (80.3%)	51 (52.6%)	38 (55.9%)	39 (60.0%)	*p*<0.00001
**Timelapse incubator (TLT)**	Amber	211 (48.2%)	122 (58.6%)	42 (43.2%)	26 (38.2%)	27 (41.5%)	*p*<0.005
**Hyaluronate**	Amber	206 (47.0%)	121 (58.2%)	34 (35.1%)	26 (38.2%)	25 (38.5%)	*p*<0.0005
**Assisted-hatching (AHA)**	Red	372 (85.1%)	196 (94.2%)	76 (78.4%)	51 (75.0%)	49 (75.4%)	*p*<0.00001
**Chronic endometritis examination**	Red	338 (77.2%)	176 (84.6%)	57 (58.8%)	52 (76.5%)	53 (81.5%)	*p*<0.00001
**Chronic endometritis treatment**	Red	337 (76.9%)	179 (86.1%)	57 (58.8%)	49 (72.1%)	52 (80.0%)	*p*<0.00001
**Endometrial receptivity array (ERA)**	Red	281 (64.2%)	165 (79.3%)	53 (54.6%)	39 (57.4%)	24 (36.9%)	*p*<0.00001
**Endometrial microbiome metagenomic analysis (EMMA)**	Red	258 (58.9%)	148 (71.2%)	46 (47.4%)	40 (58.8%)	24 (36.9%)	*p*<0.00001
**Two-step embryo transfer**	Red	214 (48.9%)	120 (57.7%)	45.4 (44)	25 (36.8%)	25 (38.5%)	*p*<0.005
**Helper-T (Th)1/Th2 cell ratio test**	Red	194 (44.3%)	117 (56.3%)	40 (41.2%)	22 (32.4%)	15 (23.1%)	*p*<0.00001
**Endometrial injection of embryo culture supernatant**	Red	173 (39.5%)	105 (50.5%)	33 (34%)	21 (30.9%)	14 (21.5%)	*p*<0.00001
**Immunosuppressant agent treatment**	Red	142 (32.4%)	93 (44.7%)	30 (30.9%)	15 (22.1%)	4 (6.2%)	*p*<0.00001
**Endometrial scratching**	Red	116 (26.5%)	70 (33.6%)	17 (17.5%)	12 (17.6%)	17 (26.2%)	*p*<0.01
**Platelet-Rich Plasma (PRP) intrauterine infusion**	Red	97 (22.1%)	65 (31.2%)	19 (19.6%)	11 (16.2%)	2 (3.1%)	*p*<0.00001
**Preimplantation genetic testing for aneuploidy (PGT-A)**	Red	93 (21.2%)	54 (26.0%)	15 (15.5%)	8 (11.8%)	16 (24.6%)	*p*<0.05
***In vitro* maturation (IVM)**	Red	76 (17.4%)	45 (21.6%)	14 (14.4%)	5 (7.4%)	12 (18.5%)	*p*<0.05
**G-CSF administration**	Red	71 (16.2%)	46 (22.1%)	14 (14.4%)	8 (11.8%)	3 (4.6%)	*p*<0.005
**Intracytoplasmic morphologically selected sperm injection (IMSI)**	Red	68 (15.5%)	44 (21.2%)	12 (12.4%)	6 (8.8%)	6 (9.2%)	*p*<0.05

Values are presented as % (n/total), unless otherwise indicated. Statistical significance is set at p<0.05.

The add-on treatments with “variations” in adoption rates were ERA, EMMA, Th1/Th2 cell ratio test, endometrial injection of embryo culture supernatant, immunosuppressive agents, and PRP therapy (**Table 1**). These were commonly associated with high adoption rates in outpatient clinics and low adoption rates in university hospitals.

### 3.3 Cost of IVF add-on treatment

We examined the extent to which an add-on treatment was provided according to facility type in Japan. Medical care that requires human and medical resources may incur high costs. Nevertheless, several add-on treatments were provided at no extra cost, regardless of facility type, including chronic endometritis treatment, TLT, IVM, and IMSI. One facility, classified as a outpatient clinic, was found to charge 400,000 yen (2697.5 USD, as of September 23, 2023) for IVM. These results highlight the unique characteristics of the IVF add-on supplement system in Japan (**Table 2**).

**Table 2:** Cost of *in vitro* fertilization add-on treatments according to facility.

	Rating in the current paper	Total	Outpatient Clinic	Inpatient Clinic	Hospital	University Hospital	
		(n=438)	(n=208)	(n=97)	(n=68)	(n=65)	*p* value
**Artificial Oocyte Activation (AOA)**	Amber	12,000 (0-20,000)	16,500 (0-22,000)	15,000 (0-20,000)	0 (0-10,000)	0 (0-19,000)	*p*<0.01
**Timelapse incubator (TLT)**	Amber	0 (0-20,000)	0 (0-10,000)	0 (0-0)	0 (0-0)	0 (0-0)	*p*=0.65
**Hyaluronate**	Amber	0 (0-11,000)	4,180 (0-15,000)	0 (0-6,700)	0 (0-10,000)	0 (0-11,250)	*p*=0.14
**Assisted-hatching (AHA)**	Red	20,000 (10,000-25,000)	20,000 (15,000-27,875)	15,750 (5,000-22,550)	20,000 (6,250-26,500)	19,000 (0-22,000)	*p*<0.05
**Chronic endometritis examination**	Red	10,000 (4,000-15,500)	11,000 (1,875-18,000)	4,000 (0-10,750)	5,000 (0-21,000)	0 (0-10,000)	*p*=0.19
**Chronic endometritis treatment**	Red	0 (0-30,00)	0 (0-3,000)	0 (0-2,575)	0 (0-3,000)	0 (0-2,000)	*p*=0.66
**Endometrial receptivity array (ERA)**	Red	120,000 (100,000-136,375)	125,000 (110,000-140,000)	110,000 (100,000-138,000)	120,000 (95,750-132,750)	110,330 (100,000-120,000)	*p*=0.13
**Endometrial microbiome metagenomic analysis (EMMA)**	Red	50,000 (40,000-66,000)	50,000 (40,000-66,000)	50,000 (40,000-61,000)	50,000 (35,000-65,000)	50,000 (40,000-65,515)	*p*=0.70
**Two-step embryo transfer**	Red	30,000 (10,000-50,000)	30,000 (20,000-54,930)	30,000 (10,000-46,500)	22,500 (7,500-37,250)	10,000 (0-31,000)	*p*<0.05
**Helper-T (Th)1/Th2 cell ratio test**	Red	15,000 (10,000-20,000)	15,000 (10,000-21,587)	15,150 (5,500-12,000)	10,000 (5,750-20,000)	13,750 (10,250-19,000)	*p*=0.41
**Endometrial injection of embryo culture supernatant**	Red	20,000 (10,000-27,500)	20,000 (11,085-30,000)	15,000 (10,000-20,000)	20,000 (13,000-25,000)	0 (0-12,500)	*p*<0.01
**Immunosuppressant agent treatment**	Red	1,500 (0-10,000)	1,000 (0-10,000)	2,500 (0-12,000)	5,000 (2,000-10,000)	3,000 (1,500-6,500)	*p*=0.55
**Endometrial scratching**	Red	2,500 (0-5,000)	2,500 (0-7,300)	3,650 (2,000-5,125)	700 (0-4,000)	0 (0-6,250)	*p*=0.28
**Platelet-Rich Plasma (PRP) intrauterine infusion**	Red	165,000 (142,250-200,000)	178,300 (150,000-200,000)	150,000 (95,000-182,500)	162,500 (142,500-200,000)	200,000 (200,000-200,000)	*p*=0.09
**Preimplantation genetic testing for aneuploidy (PGT-A)**	Red	80,000 (65,750-90,000)	86,000 (70,000-90,000)	70,000 (55,000-95,000)	75,000 (60,202-82,000)	67,500 (59,300-82,500)	*p*=0.29
***In vitro* maturation (IVM)**	Red	0 (0-22,000)	0 (0-0)	0 (0-22,000)	0 (0-55,000)	0 (0-52,500)	*p*=0.74
**G-CSF administration**	Red	20,000 (15,000-30,000)	23,600 (16,125-30,000)	15,500 (12,250-21,500)	25,000 (17,500-30,000)	20,000 (15,000-20,000)	*p*=0.68
**Intracytoplasmic morphologically selected sperm injection (IMSI)**	Red	0 (0-0)	0 (0-5,000)	0 (0-0)	0 (0-0)	0 (0-0)	*p*=0.77

Values are presented as median (IQR) unless otherwise indicated. Statistical significance is set at p<0.05.

Medical care should be provided at the same price regardless of the facility. Nevertheless, IVF add-ons, including AHA, AOA, two-step embryo transfer, and endometrial injection of embryo culture supernatant, were found to have significant differences in cost according to facility type. All these add-on treatments were significantly more expensive in outpatient clinics than in university hospitals (**Table 2**).

### 3.4 Outpatient clinics are actively incorporating IVF add-on treatments

**Figure 2:**
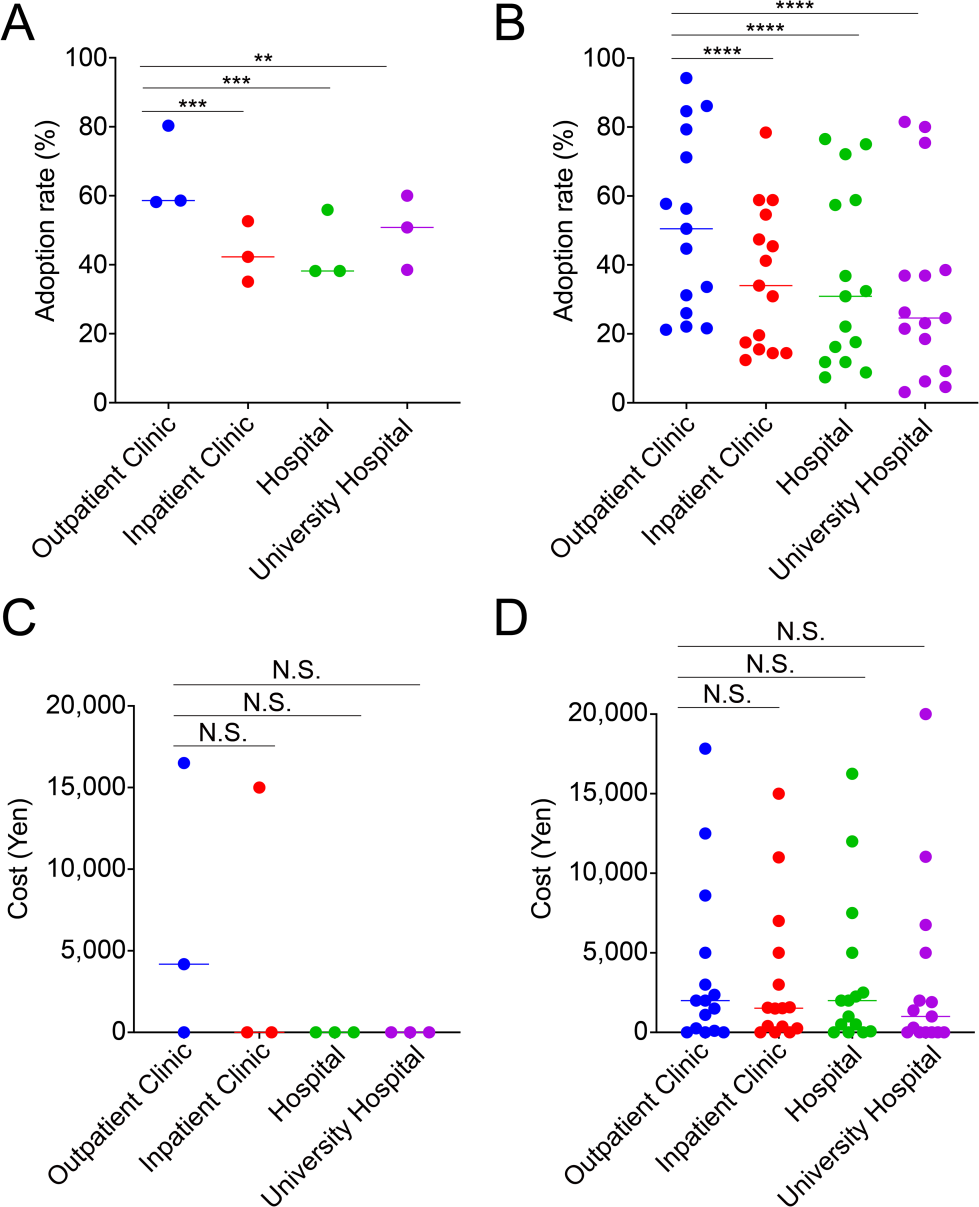
Outpatient clinics adopt significantly more add-on treatments than other facilities, but the median costs are comparable. The adoption rate of *in vitro* fertilization add-on treatments is rated as Amber **(A)** and Red **(B)**, and the cost is rated as Amber **(C)** and Red **(D)**, based on facility type. ****p<0.0001, ***p<0.001, **p<0.01, N.S., not significant.

It seems evidence-based medicine is emphasized more by university facilities emphasize than by general clinical facilities. Therefore, we examined whether there was a correlation between the evidence level and adoption rate and cost of add-ons among the four attributes. Contrary to expectations, the adoption rate of add-on treatments classified as Amber was significantly lower in university hospitals than in outpatient clinics (*p*<0.01) (**Figure 2A**). Furthermore, the add-ons rated as Red had significantly higher adoption rates at outpatient clinics than at any other facility (*p*<0.0001, each) (**Figure 2B**). In contrast, there was no significant difference in cost between facility types for both Amber and Red categories (**Figures 2C, D**).

### 3.5 Evidence levels, adoption rates, and cost of IVF add-ons

**Figure 3:**
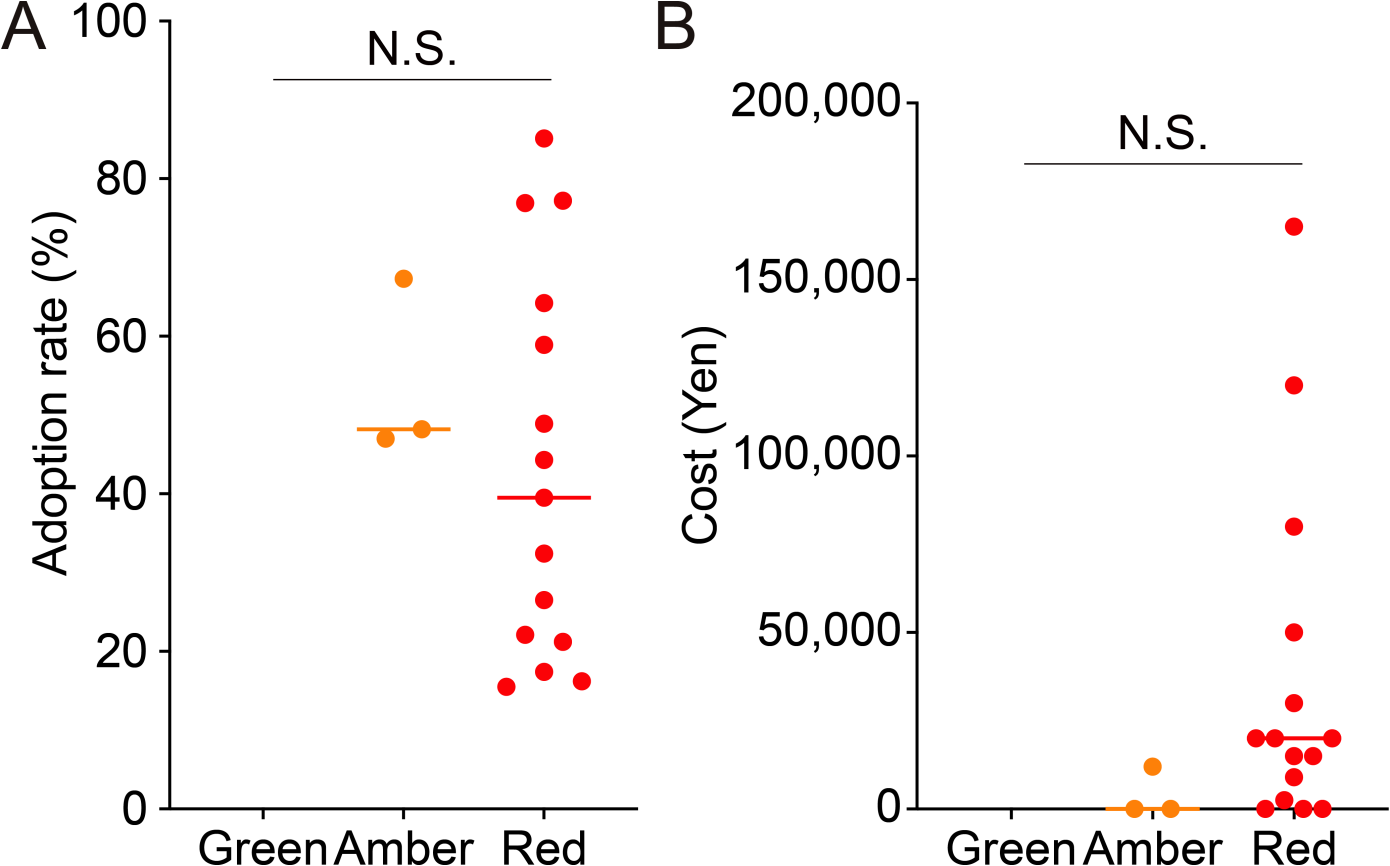
Correlation of evidence level with adoption rate and median cost of *in vitro* fertilization add-on treatments. The figure shows the adoption rate **(A)** and cost **(B)** of *in vitro* fertilization add-on treatments according to the evidence level. N.S., not significant.

We hypothesized that as the evidence levels of IVF add-ons increase, adoption rates increase and costs decrease. Adoption rates and costs were analyzed to determine whether they correlated with the evidence levels of each individual IVF add-on treatment. The median adoption rate of Amber (48.2%) was higher than that of Red (32.4%) (**Figure 3A**). The wide variability of Red compared with Amber suggests that there are large differences in the adoption rates of add-on treatments according to facility type. The median cost of Red (20,000 yen, equivalent to 134.8 USD at the exchange rate as of September 23, 2023) was higher than that of Amber (0 yen, 0 USD) (**Figure 3B**). Furthermore, the wide range of minimum to maximum values for Red indicates that there are large differences in costs according to the type of add-on treatment. Although there was a trend towards a positive correlation between the level of evidence for IVF add-ons and adoption rates and an inverse correlation with costs, it was not statistically significant.

### 3.6 Cost did not correlate with the adoption rate

Based on our results, the adoption rate and cost were suggested to be inversely correlated. Accordingly, a linear regression analysis was performed. Although there was an overall trend towards an inverse correlation, there was no significant difference (**Supplementary Figure 1A**). The same trend was observed when the analysis was conducted separately according to the facility type (**Supplementary Figures 1B-E**). This trend was similar when linear regression analysis was performed by categorizing the add-on introduction rate as the adoption rate (0%–33%, 34%–66%, 67%–99%) (**Supplementary Figure 2**).

## 4 Discussion

The current survey showed that outpatient clinics had the highest number of oocyte retrievals per facility per year, indicating their central role in reproductive medicine in Japan. These facilities have a greater number of embryologists and nurses but fewer obstetricians and gynecologists than others. Outpatient clinics actively adopted significantly more IVF add-ons than other ART centers, regardless of the evidence level. There was a positive relationship between the level of evidence and the adoption rate of IVF add-ons and a negative relationship between the level of evidence and the cost of add-ons.

Add-ons have a history of being originally developed to improve pregnancy outcomes. Although add-ons are important for the advancement of reproductive medicine, their implementation in clinical practice has faced criticism from various media outlets, owing to the perceived lack of sufficient evidence (4). To assess the effectiveness of add-ons in Japan, several studies have examined the efficacy of add-on treatments within the framework of advanced research coinciding with the initiation of insurance coverage (17). In addition to explaining to patients that add-ons are distinct from essential medical care, this may provide crucial evidence for determining whether add-ons constitute a more essential medical treatment by establishing a stronger evidential basis for their efficacy. However, because the medical facilities responsible for clinical research may derive benefits from add-ons, caution should be exercised when interpreting the results, considering potential conflicts of interest.

In addition to the effectiveness and safety of each medical treatment, it is important to improve cost-effectiveness and patient satisfaction. The current study showed that the accumulation of evidence on the effectiveness and safety of add-on treatments may lead to an increasing adoption rate, which would produce scale merit and reduce costs. Additionally, as represented by next-generation sequencing, technological advances may lead to decreased costs (18). High-quality medical care is expected to be more cost-effective, and a positive cycle of increasing adoption rates and accumulating evidence is expected to emerge.

In contrast, there were no significant differences between evidence levels, adoption rates, and costs. There are three reasons for this: (i) The uniqueness of the clinics: Outpatient clinics adopted a significantly higher number of add-ons than other facility types, regardless of the evidence level. They may anticipate gain from the publicity effect of introducing “novel” and “costly” tests and treatments (19). Clinics have more freedom than university hospitals to offer commercially available treatments, even those that have not been adequately proven to be safe or effective. (ii) Cost-free add-on treatments, e.g., TLT: The median cost of TLT in the current study was 0 yen (0 USD), regardless of facility type, even though TLT is considered expensive (20), with an initial implementation cost of hundreds and thousands of USD (21). According to a French survey of embryologists, the reasons for not implementing TLT were high initial implementation costs (50%) and a lack of data to support its clinical usefulness (37.5%) (22), as proven by a recent RCT trial (23). One reason for the discrepancy between the actual situation in the United States, France, and Japan is that the standardization of TLT, which facilitates the expansion of embryo culture capacity (24), may lead to the loss of the option of culture methods with a conventional incubator, limiting patients’ ability to bear additional costs. There is a concern that cost-free add-on treatments could be attributed to financial constraints that prevent payment of salaries to healthcare providers, which could result in a shortage of staff able to provide adequate support in the decision-making process. Because of the free pricing available under free treatment, facilities that do not incur add-on costs may add these costs to the normal ART costs. (iii) Limited evidence of only three treatments classified as Amber: Add-ons that allow medical facilities to control costs only to a limited extent are likely to have a significant impact on outcomes. In cases where no significant differences between different facilities were found, such as ERA, EMMA, analysis of infectious chronic endometritis, PRP, and PGT-A, costs are determined by the outsourcing company. In particular, the environment surrounding PGT-A has undergone significant changes over the past 25 years. The substantial expenses associated with the equipment required for PGT-A and the potential for cost reduction through batch processing of samples have led companies to offer genetic services to multiple IVF clinics (25). Therefore, the absence of variation in the additional cost of PGT-A based on facility type is because of the commonality of the contractors involved. In contrast, AHA, AOA, and two-step embryo transfer showed significant differences in overall costs, but their pricing was left to the discretion of the facility.

One of the strengths of the current study is that it relies on a cost survey of healthcare providers, which may represent actual costs more accurately than previous studies on patients (3). In addition, the response rate to the survey was >70%, which is the highest response rate ever recorded in Japan (51%–63% in a survey conducted by the Ministry of Health, Labour and Welfare in 2020). This high response rate appropriately represents the actual status of ART in Japan before insurance coverage.

### 4.1 Limitation

As this survey was conducted in medical facilities, the extent to which patients choose add-ons and the total cost paid for reproductive health-related services were unclear. In addition, a few add-ons were classified as Amber, which limits the statistical analysis.

### 4.2 Conclusion

This survey of ART facilities in Japan showed that IVF add-ons are widespread and that the use of some add-ons creates a significant financial burden. Although numerous add-ons are available for IVF, their efficacy in improving pregnancy outcomes has not been scientifically demonstrated. Add-ons that have strong scientific evidence regarding safety and efficacy are widely adopted, regardless of facility type, whereas costly add-ons have low adoption rates because of the emphasis placed by healthcare professionals on scientific evidence. In Japan, some add-ons have been made eligible for national health insurance coverage from 2022 onwards; however, concurrent collaborative research by the Japan Society of Obstetrics and Gynaecology and the Japan Society for Reproductive Medicine as well as performance evaluations based on advanced medical care are being conducted to assess the safety and efficacy of add-ons. Based on these results, a review of insurance medical care is planned for fiscal year 2024. This survey played an important role in the formation of consensus. Additionally, it is expected to be a decision-making tool for clinicians and patients who suffer from infertility and consider add-ons as medical treatments.

## Data Availability

The raw data supporting the conclusions of this article will be made available by the authors, without undue reservation.

## Supplementary material

Supplementary Figure 1 Correlation of adoption rate with the median cost of *in vitro* fertilization add-on treatments. Linear regression of all facility types **(A)**, outpatient clinics **(B)**, inpatient clinics **(C)**, hospitals **(D)**, and university hospitals **(E)**.

Supplementary Figure 2 Correlations of adoption rate with the median costs of *in vitro* fertilization add-on treatments, categorized into 0%–33%, 34%–66%, and 67%–99% adoption rate. The adoption rate of *in vitro* fertilization add-on treatments (>34%) for all categories **(A)**, 0%–33% vs. 34%–66% **(B)**, 0%–33% vs. 67%–99% **(C)**, 34%–66% vs. 67%–99% **(D)**. There is no relationship between cost and the adoption rate, which is divided into three categories.
