# Supplementary figures and images for: Survey of in vitro fertilization add-ons in Japan (Izanami project)
Source: Front Endocrinol (Lausanne). 2024 Oct 8;15:1404601. doi: 10.3389/fendo.2024.1404601 (PMC11493599; doi:10.3389/fendo.2024.1404601)

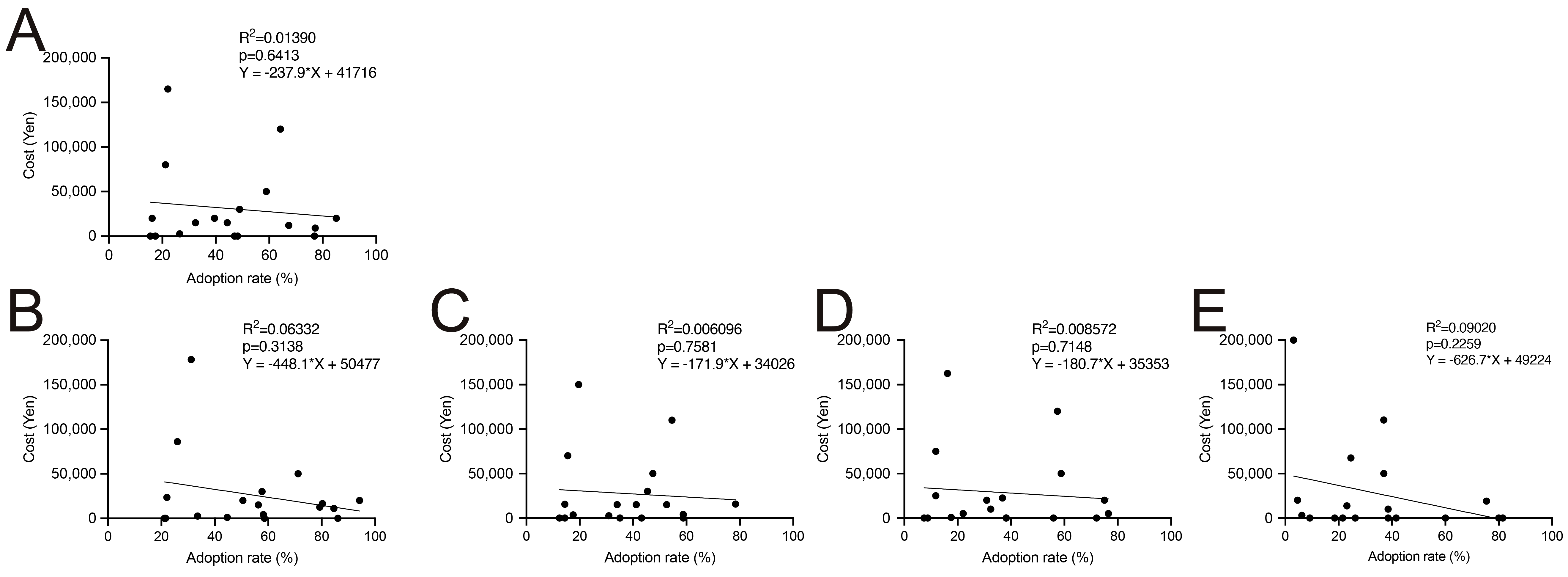

Supplement: Supplementary Figure 1 — Correlation of adoption rate with the median cost of in vitro fertilization add-on treatments. Linear regression of all facility types (A), outpatient clinics (B), inpatient clinics (C), hospitals (D), and university hospitals (E). [file Image1.tif]

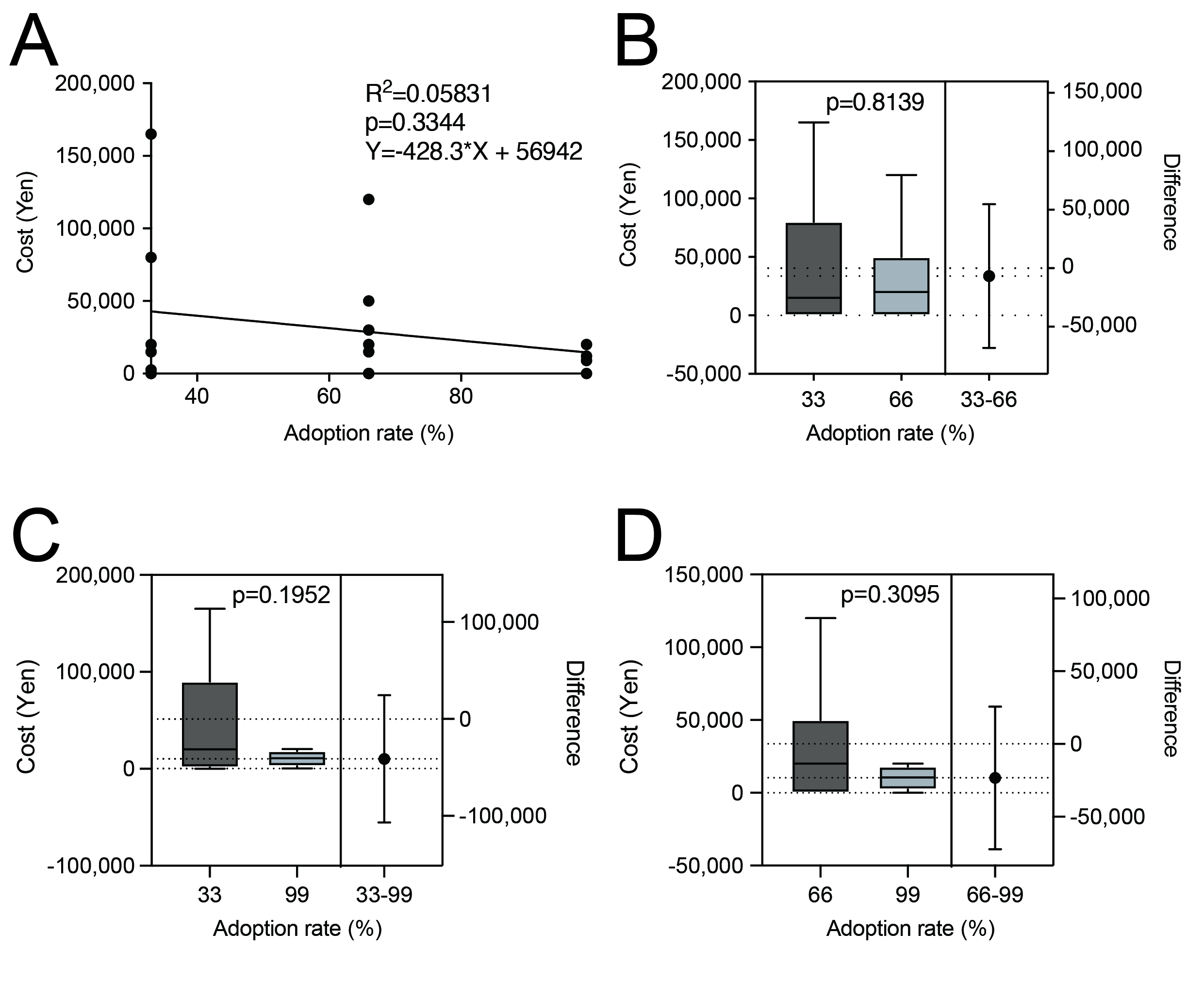

Supplement: Supplementary Figure 2 — Correlations of adoption rate with the median costs of in vitro fertilization add-on treatments, categorized into 0%–33%, 34%–66%, and 67%–99% adoption rate. The adoption rate of in vitro fertilization add-on treatments (>34%) for all categories (A), 0%–33% vs. 34%–66% (B), 0%–33% vs. 67%–99% (C), 34%–66% vs. 67%–99% (D). There is no relationship between cost and the adoption rate, which is divided into three categories. [file Image2.tif]
